# Human adenovirus Coinfection aggravates the severity of *Mycoplasma pneumoniae* pneumonia in children

**DOI:** 10.1186/s12879-020-05152-x

**Published:** 2020-06-16

**Authors:** Jiaojiao Gao, Lili Xu, Baoping Xu, Zhengde Xie, Kunling Shen

**Affiliations:** National Clinical Research Center for Respiratory Diseases, Key Laboratory of Major Diseases in Children, Ministry of Education, Beijing Key Laboratory of Pediatric Respiratory Infection diseases, Research Unit of Critical Infection in Children, Chinese Academy of Medical Sciences, 2019RU016, Laboratory of Infection and Virology, Beijing Pediatric Research Institute, Beijing Children’s Hospital, Capital Medical University, National Center for Children’s Health, Beijing, China

**Keywords:** *Mycoplasma pneumoniae*, Adenovirus, Pneumonia, Case-control study, Children

## Abstract

**Background:**

*Mycoplasma pneumoniae* (*M. pneumoniae*) is an important pathogen of community-acquired pneumonia (CAP) in children. The coinfection rate of *M. pneumoniae* pneumonia (MPP) can reach 52% in some areas, but the effects of coinfection with different pathogens have not been clearly recognized.

**Methods:**

The cases of MPP hospitalized in Beijing Children’s Hospital from 1/1/2014 to 12/31/2016 were screened. MPP patients coinfected with Human adenovirus (HAdV) were categorized into the research group. Patients with single *M. pneumoniae* infection were categorized into the control group, matching the research group by age and admission time with a ratio of 1:3. Clinical manifestations, laboratory examinations, and disease severity were compared between these two groups.

**Results:**

A total of 2540 hospitalized MPP cases were screened in Beijing Children’s Hospital, among which thirty cases were enrolled in the research group and ninety cases were enrolled in the control group. The results indicated that patients in the research group had longer hospital stays, longer fever durations and a higher rate of dyspnea, as well as a larger proportion applications of oxygen therapy and noninvasive continuous positive airway pressure (NCPAP). No obvious differences were found in lab examinations within the two groups. Regarding disease severity, the proportions of extremely severe pneumonia and severe disease defined by the clinical score system were higher in the research group than in the control group.

**Conclusion:**

Compared with single *M. pneumoniae* infection, MPP coinfected with HAdV in children was relatively more serious.

## Background

*Mycoplasma pneumoniae* (*M. pneumoniae*) is an important pathogen of community-acquired pneumonia (CAP) in hospitalized children. *M. pneumoniae* circulates throughout a year, and there is an epidemic of MP every 3–7 years [1]. During the epidemic, *M. pneumoniae* is responsible for 20–40% of community-acquired bacterial pneumonia [2]. A study of respiratory pathogens in children with lower respiratory tract infections from Shanghai showed that *M. pneumoniae* was the most common pathogen from 2013 to 2015 [3]. In addition, the detection rate of *M. pneumoniae* in pediatric CAP had a positive correlation with the increase in the age of the children [4].

*M. pneumoniae* infection can be self-limited [5], but it can also lead to severe pneumonia [6, 7] and even acute respiratory distress syndrome (ARDS) in children. *M. pneumoniae* infection can also cause many extrapulmonary manifestations [8, 9], which makes diagnosis and treatment more difficult. In addition, *M. pneumoniae* can damage the epithelial cells and cilia of the human airway, affect the function of the mucus-ciliary clearance system, and affect host immune function. The coinfection rate can reach 52% in *Mycoplasma pneumoniae* pneumonia (MPP) [10], but the effects of coinfection with other pathogens on the clinical features of MPP have not been clearly recognized.

Human adenovirus (HAdV) is an important pathogen of respiratory tract infection in children and is responsible for 4–10% of pediatric CAP [11]. To elucidate the impact of HAdV coinfection on the clinical manifestation of MPP in children, we performed this case-control study.

## Methods

### Study population

The cases of MPP hospitalized in Beijing Children’s Hospital from 1/1/2014 to 12/31/2016 were screened. MPP patients coinfected with HAdV were categorized into the research group. For each patient in the research group, we matched three cases with single *M. pneumoniae* infection as controls. The control group was matched to the research group by age and admission period, suggesting that every child in the control groups should be in the same age group as the patient in the research group, and these patients were collected within 2 weeks before and 2 weeks after the coinfection cases. The nearest cases were recruited first. In detail, the patients were divided into four age groups: < 2 years old, 2–5 years old, 5–10 years old, and > 10 years old.

### Inclusion and exclusion criteria

The inclusion criteria for the research group were as follows: 1) clinical manifestations of pneumonia, such as fever, cough and radiographic findings of pneumonia; 2) evidence of *M. pneumoniae* infection: serological *M. pneumoniae* antibody titer ≥1:160 or a positive result of *M. pneumoniae* RNA of oropharyngeal swabs with real-time polymerase chain reaction (PCR); and 3) evidence of HAdV infection: a positive result of HAdV-DNA from blood or respiratory samples or a positive result of HAdV antigen from nasopharyngeal aspirates.

Control subjects should meet the first and second inclusion criteria of the research group and have a negative result after HAdV examination.

Children with any of the following factors were excluded: 1) neonatal pneumonia; 2) coinfected with other pathogens confirmed by laboratory tests; 3) convalescent pneumonia; 4) long-term use of glucocorticoids or immunosuppressive agents; and 5) congenital heart or chronic lung disease.

If a patient in the research group received recurrent fever after more than 3 days of normal temperature and HAdV infection was confirmed at the same time, then he or she was diagnosed with nosocomial infection.

### Clinical information collection

The clinical manifestations, laboratory examinations, imaging characteristics, and disease severity were compared between these two groups. Three methods were used to evaluate the severity of disease. 1) According to the national guidelines for pediatric CAP in China, pneumonia was divided into mild and severe (Table 1). 2) According to the same guidelines, extremely severe pneumonia was diagnosed if the child presented with central cyanosis, severe respiratory distress, refusal to eat, dehydration or altered consciousness (somnolence, coma, convulsions), and he or she experienced extremely severe pneumonia. 3) Based on the clinical scoring system designed by Carmen L. Larranaga and other scholars to classify the severity of acute lower respiratory tract infection caused by respiratory syncytial virus, we divided pneumonia into mild, moderate and severe grades (Table 2) [12].

**Table 1:** Disease Severity Assessment of CAP in Children

Clinical manifestations	Mild CAP	Severe CAP
**General situation**	Good	Not good
**Refusal to eat or dehydration**	No	Yes
**Disorder of consciousness**	No	Yes
**Respiratory rate**	Normal or slightly increase	Significantly increase^a^
**Cyanosis**	No	Yes
**Dyspnea**	No	Yes
**Multilobar infiltrates**	No	Yes
**Pleural effusion**	No	Yes
**Oxygen saturation**	> 0.96	≤0.92
**Extrapulmonary manifestations**	No	Yes
**Assessment**	Present all above manifestations	Present any of the above manifestations

^a^Significantly increased respiratory rate: infants > 70 bpm, older children > 50 bpm

**Table 2:** Clinical Scoring System

Factor	Score				Maximal Score^**c**^
	0	1	2	3	
**Hospitalization**	No	≤5 d	> 5 d	PICU^a^(+ 1/2)	0–5
**Supplemental oxygen**	No	≤1 d	1–3 d(+ 1)	> 3 d(+ 1)	0–4
**Max FiO**_**2**_**(%)**	21	22–30	≥31	MV ^b^(+ 2)	0–5

^a^*PICU* Pediatric intensive care unit

^b^*MV* Mechanical ventilation

^c^A total score value of 7 or greater was defined as severe disease, and less than 7 were considered as mild or moderate disease.

The extrapulmonary manifestations involved in our study included mucocutaneous manifestations, myocardial damage and liver damage. Myocardial damage includes abnormal Electrocardiogram (ECG) and Creatine kinase isoenzyme (CK-MB) above the upper limit of normal (25 U/L). Liver damage refers to glutamic-pyruvic transaminase (ALT) above the upper limit of normal (40 U/L).

### Statistical analysis

Statistical analysis was performed with SPSS Statistics (v21, IBM Corp., USA). The independent sample t-test and two independent sample rank sum test were used to compare continuous variables between two groups. Pearson’s chi-square or Fisher’s exact test was used for categorical data. *P* ≤ 0.05 was considered to be statistically significant.

## Results

### Patients enrollment

From 2014 to 2016, 2540 hospitalized MPP cases were found in Beijing Children’s Hospital, and 53 cases were diagnosed with HAdV coinfection. However, 11 of these cases were excluded: 4 cases had insufficient lab examinations, 5 cases had coinfection with other pathogens, 1 case had convalescent pneumonia, and 1 case had congenital heart disease. Among the remaining 42 coinfection cases, 12 cases could not be included because no matching cases were found. Eventually, 30 cases were included in the research group, and 90 cases were included in the control group.

### Demographic characteristics of the patients

Of the 30 research cases, 2 cases were < 2 years old, 14 cases were 2–5 years old, 11 cases and 3 cases were 5–10 years old and > 10 years old, respectively. Most of these patients visited our hospital in autumn and winter.

Boys accounted for 67% in the research group and 50% in the control group (Table 3).

**Table 3:** Clinical Characteristics between the Research Group and the Control Group

Characteristic	Control group (***n*** = 90)	Research group (***n*** = 30)	***P*** value
**Males n (%)**	45 (50%)	20 (67%)	0.084
**Hospital stay (day), median**	8 (3–15)	10 (3–33)	0.001
**Fever days before admission, median**	7 (2–46)	7.5 (3–25)	0.751
**Fever duration (day), median**	9 (2–31)	14 (7–28)	<0.01
**Max temperature (°C), median**	39.50 (37.8–41.0)	39.55 (38.0–40.5)	0.474
**Heart rate (bpm), mean**	110 (80–142)	116 (88–155)	0.043
**Respiratory rate (bpm), median**	24 (18–44)	24 (20–56)	–
**Disturbance of consciousness, n**	0	1 (3.3%)	0.25
**Cyanosis, n**	1	0	–
**Dyspnea, n**	2 (2.2%)	7 (23.3%)	0.001
**Abnormal breath sounds, n**	55 (61.1%)	19 (63.3%)	0.503

### Comparisons of clinical characteristics

The clinical characteristics of the two groups are shown in Table 3. The research group had a longer hospital stay (10 days vs 8 days, *P* = 0.001), longer fever duration (14 days vs 9 days, *P* < 0.01), increased heart rate (116 bpm vs 110 bpm, *P* = 0.043) and a higher rate of dyspnea (23.3% vs 2.2%, *P* = 0.001).

### Comparisons of laboratory and radiographic findings

No difference was found in laboratory examination and radiographic findings between the two groups, and the proportion of extrapulmonary manifestations was similar between the two groups (Table 4).

**Table 4:** Laboratory and Radiographic Findings between the Research Group and the Control Group

Characteristics	Control group (***n*** = 90)	Research group (***n*** = 30)	***P*** value
**Leukocyte count (× 10^9/L), median**	7.355 (2.9–20.4)	6.995 (3.8–20.9)	0.833
**Neutrophil (%), mean**	58.8 ± 12.9 (26.4–86.2)	59.4 ± 12.1 (31.0–86.0)	0.805
**Lymphocyte (%), mean**	30.8 ± 12.1 (8.9–64.9)	31.1 ± 10.7 (9.9–56.4)	0.880
**Anemia, n**	14 (15.6%)	7 (23.3%)	0.239
**Platelet (×10^9/L), median**	305 (90–680)	264 (111–644)	0.01
**C-reactive protein> 8 mg/L, n**	51 (56.7%)	16 (53.3%)	0.456
**Elevated serum CK-MB, n**	2 (2.2%)	2 (6.7%)	0.263
**Abnormal ECG, n**^**a**^	36 (46.7%)	10 (45.5%)	0.555
**Decrease PaO**_**2**_**, n**^**b**^	0	0	–
**LDH (U/L), median**	322 (172–1820)	348 (190–693)	0.337
**Elevated serum PCT, n**	43 (47.8%)	18 (60%)	0.407
**Multilobar infiltrates, n**	61 (67.8%)	21 (70%)	0.506
**Pleural effusion, n**	23 (25.6%)	8 (26.7%)	0.54
**Rash, n**	6 (6.7%)	1 (3.3%)	0.44
**Myocardial damage, n**	37 (41.1%)	11 (36.7%)	0.418
**Liver damage, n**	12 (13.3%)	1 (3.3%)	0.112

^a^The ECG results were observed for 77 cases in the control group and 22 in the research group

^b^The results of the arterial blood gas analysis were found for 51 cases in the control group and 23 cases in the research group

Although the platelet count was higher in the research group, the data in both groups were within the normal reference range for children.

### Comparisons of treatment

For treatment, more than half of the children in both groups received fiberoptic bronchoscopy, compared with 53.3% in the research group and 65.6% in the control group, and the difference was not statistically significant (Table 5).

**Table 5:** Treatment and Disease Severity between the Research Group and Control Group

Characteristic, n (%)	Control group (***n*** = 90)	Research group (***n*** = 30)	P value
**Fiberoptic bronchoscopy**	59 (65.6%)	16 (53.3%)	0.278
**Oxygen therapy**	58 (64%)	28 (93%)	0.001
**NCPAP**	0	5 (16.7%)	0.01
**Invasive mechanical ventilation**	0	0	–
**PICU admission**	0	0	–
**Readmission**	9 (10%)	3 (10%)	–
**Severe CAP**	72 (80%)	26 (87%)	0.301
**Extremely severe pneumonia**	3 (3.3%)	7 (23.3%)	0.002
**Severe disease defined by clinical score system**	13 (14.4%)	14 (46.6%)	0.001

No patients were treated with invasive mechanical ventilation or transferred to the PICU. However, our study showed that the proportion of oxygen therapy (93% vs 64%, *P* = 0.001) and noninvasive continuous positive airway pressure (NCPAP) (16.7% vs 0, *P* = 0.01) was higher in the research group than in the control group (Table 5).

The readmission rate was 10% in both groups. Three patients in the research group and eight in the control group were readmitted for fiberoptic bronchoscopy, and one patient in the control group returned to the hospital because of bronchiolitis obliterans.

### Comparisons of the disease severity

In terms of disease severity, the proportions of severe CAP in the research group and the control group were 87 and 80%, respectively, and the difference was not statistically significant. However, the proportion of extremely severe pneumonia in the research group (23.3%) was higher than that in the control group (3.3%) (*P* = 0.002). According to the clinical scoring system, the severe proportions in the research group and the control group were 46.6 and 14.4% (*P* = 0.001), respectively (Table 5).

### Nosocomial infection and nonnosocomial infection cases

Further analysis of nosocomial infection was performed according to the time of diagnosis of HAdV infection.

Nine children in the research group were nosocomial infection cases. Comparing these 9 children with their neighbors, the difference was consistent with the total research group and the control group (Table 6).

**Table 6:** Comparisons of Nosocomial Infection Cases in the Research Group and Their Matched Controls

Characteristics	Control group (***n*** = 27)	Research group (***n*** = 9)	P value
**Hospital stay (day), median**	7 (3–13)	19 (9–33)	0.002
**Fever days before admission, median**	7 (2–40)	7 (5–12)	–
**Fever duration (day), median**	8 (2–31)	19 (12–28)	0.001
**Max temperature (°C), mean**	39.32 ± 0.67	39.18 ± 0.62	0.571
**Cyanosis, n (%)**	0	0	–
**Dyspnea, n (%)**	1 (3.7%)	3 (33.3%)	0.041
**Fiberoptic bronchoscopy**	20 (74.1%)	7 (77.8%)	0.602
**Oxygen therapy**	16 (59.3%)	9 (100%)	0.022
**NCPAP**	0	3 (33.3%)	0.012
**Readmission**	3 (11.1%)	1 (11.1%)	0.745
**Severe CAP**	23 (85.2%)	9 (100%)	0.298
**Extremely severe pneumonia**	0%	3 (33.3%)	0.012
**Severe disease defined by clinical score system**	5 (18.5%)	5 (55.6%)	0.046

The remaining 21 co-infected children in the research group were admitted to the hospital with adenovirus coinfection. When compared with their neighbors, no significant differences were found in hospital stay, NCPAP use and the proportion of extremely severe pneumonia between the two groups (Table 7).

**Table 7:** Comparisons of Nonnosocomial Infection Cases in the Research Group and Their Matched Controls

Characteristics	Control group (***n*** = 63)	Research group (***n*** = 21)	P value
**Hospital stay (day), median**	8.0 (3–15)	9.0 (3–12)	0.069
**Fever days before admission, median**	7 (0–46)	9 (3–21)	0.449
**Fever duration (day), median**	9 (2–29)	12 (7–26)	0.028
**Max temperature (°C), median**	39.5 (37.8–41.0)	39.8 (38.0–40.5)	0.255
**Heart rate (bpm), mean**	112 ± 13 (80–142)	117 ± 14 (88–148)	0.134
**Respiratory rate (bpm), median**	25 (18–44)	25 (21–56)	–
**Disturbance of consciousness, n**	0	1 (4.8%)	0.25
**Cyanosis, n**	1 (1.6%)	0	0.75
**Dyspnea, n**	1 (1.6%)	4 (19%)	0.013
**Abnormal breath sounds, n**	39 (61.9%)	14 (66.7%)	0.797
**Leukocyte count (× 10^9/L), median**	7.49 (2.9–20.42)	5.95 (3.78–13.88)	0.614
**Anemia, n**	11 (17.5%)	6 (28.6%)	0.213
**Platelet (× 10^9/L), mean**	323 ± 117 (90–622)	238 ± 70 (111–342)	< 0.001
**C-reactive protein> 8 mg/L, n**	34 (54.0%)	12 (57.1%)	0.502
**Elevated serum CK-MB, n**	2 (3.2%)	2 (9.5%)	0.264
**Multilobar infiltrates, n**	42 (66.7%)	13 (61.9%)	0.442
**Pleural effusion, n**	17.0 (27%)	7 (33.3%)	0.383
**Rash, n**	4 (4.3%)	0	0.309
**Myocardial damage, n**	22 (34.9%)	6 (28.6%)	0.4
**Liver damage, n**	9 (14.3%)	1 (4.8%)	0.226
**Fiberoptic bronchoscopy**	39 (61.9%)	9 (42.9%)	0.102
**Oxygen therapy**	38 (60.3%)	19 (90.5%)	0.008
**NCPAP**	0	2 (9.5%)	0.06
**Readmission**	6 (9.5%)	2 (9.5%)	0.683
**Severe CAP**	49 (77.8%)	17 (81.0%)	0.512
**Extremely severe pneumonia**	3 (4.8%)	4 (19%)	0.062
**Severe disease defined by clinical score system**	8 (12.7%)	9 (42.9%)	0.005

## Discussion

Compared with *M. pneumoniae* mono-infection, patients coinfected with HAdV had a longer hospital stay and fever duration and a higher rate of dyspnea, which led to a higher rate of oxygen therapy and NCPAP use, as well as a higher proportion of extremely severe pneumonia and severe disease defined by the clinical score system.

There was no similar study on the effect of coinfection with HAdV in MPP, but there are some studies on MPP coinfections with other pathogens.

In terms of clinical manifestations, our study showed that the duration of fever was longer in the research group, regardless of the nosocomial infection status. Chen and colleagues studied 201 children with MPP in China, of which 103 children were coinfected with other pathogens, including *Chlamydia*, viruses and bacteria, and 6 of these children were coinfected with HadV. The results showed that the proportion of fever duration > 10 days (40.8%) in the mixed infection group was significantly higher than that in the nonmixed infection group (24.5%, *P* = 0.014), which was consistent with our results [13]. It is reasonable that coinfection with HAdV can prolong the clearance time of the pathogen and aggravate the host immune response, which leads to longer inflammation times. However, when Chin-Yung Chiu et al. compared the clinical manifestations of *M. pneumoniae* mono-infection (*n* = 31), *M. pneumoniae* with *Streptococcus pneumoniae* coinfection (*n* = 9) and *M. pneumoniae* with virus coinfection (*n* = 19), these authors found that the fever duration of *M. pneumoniae*- and *Streptococcus pneumoniae*-infected children was longer than that of *M. pneumoniae* mono-infected children, but there was no significant difference between *M. pneumoniae* mono-infection and virus coinfection [14]. In this study, only 7 children were coinfected with HAdV in a total of 19 virus-coinfection patients. The different types of the pathogens involved in this study may be the reason for the disparate findings. As our study only focused on HAdV coinfection, it may better reflect the effects of HAdV infection on the clinical manifestations of MPP. Furthermore, our study reduced the effects of confounding factors, such as age and admission time by matching.

We also found that the incidence of dyspnea in the research group was higher than that in the control group. Therefore, the proportions of oxygen therapy and NCPAP were higher in the research group. In 2015, Jiang et al. conducted a study on the clinical characteristics of 593 hospitalized CAP patients with pathogens confirmed in the Children’s Hospital of Soochow University. The proportions of oxygen therapy among the single bacteria group and mixed bacteria and viruses group were 2.7 and 9.5%, respectively (*P* = 0.02). These results suggested that the proportion of oxygen therapy was higher in the mixed group than in the single bacteria group. However, there was no further analysis of the specific pathogen of the mixed infections [15].

The incidence of dyspnea in HAdV-induced lower respiratory tract infection was as high as 40.7% [16]. An analysis of 213 children with severe HAdV pneumonia by Liu et al. showed that the incidence of respiratory failure was 82.2%, and 70 cases required mechanical ventilation [17]. Moreover, cases of severe pneumonia caused by HAdV requiring extracorporeal membrane oxygenation (ECMO) have been reported in Taiwan and abroad [18, 19]. In our study, none of these cases required invasive mechanical ventilation, which may be associated with early and timely oxygen therapy and NCPAP use. In combination with the results of our study and former studies, we suggest that physicians should pay attention to the possibility of HAdV coinfection in the treatment of MPP when dyspnea appears. If necessary, timely isolation can prevent nosocomial infection.

Regarding the severity of disease, we found that the proportion of extremely severe pneumonia and severe disease defined by the clinical score system was higher in the research group. The different results of different comparison methods are related to the different parameters involved. Lu et al. studied refractory *M. pneumoniae* pneumonia (RMPP) in children and found that the proportion of severe pneumonia in the coinfection group was higher than that in the mono-infection group (7.35% vs 0.46%, *P* = 0.003) [20]. Huong Ple T conducted a study on the risk factors of severe atypical pneumonia. By multivariate logistic regression analysis, coinfection with respiratory virus was found to be one of the risk factors for severe atypical CAP in children (OR = 4.36, 95% CI = 1.46–13.0, *P* = 0.008) [21], which was consistent with our findings. Interactions of pathogens contribute to aggression of the disease severity, and the specific mechanism still needs further study.

The analysis of nosocomial infections showed that the proportion of extremely severe pneumonia in the 9 nosocomial infection cases was higher than that in their matched cases, and this difference was not observed in nonnosocomial infections. These results suggested that the proportion of extremely severe pneumonia increased significantly when nosocomial HAdV infections occurred.

From November 1996 to January 1997, 13 cases of nosocomial HAdV type 7 infection were reported in a pediatric ward of a Japanese hospital. The source of infection was a 2-year-old boy with HAdV pneumonia, pleural effusions and meningitis. Thirteen children who had been in contact with this patient in the ward developed HAdV pneumonia, and all but one patient required oxygen inhalation because of hypoxemia, and two of these patients were ventilated with a respirator. Notably, 5 of the 13 patients were hospitalized with MPP before nosocomial infection [22]. Although researchers had not assessed the severity of pneumonia, the dependence of the children on oxygen therapy could still indicate the severity of the disease in children with nosocomial infections.

We believe that the infection of HAdV acquired in the hospital is equivalent to a “secondary strike”, which may aggravate the host immune response and increase the severity of the MPP. Hence, we suggest that pediatricians isolate HAdV-infected children, and the strict handwashing of medical staff is recommended to avoid serious complications of nosocomial infection in children with MPP.

This study still has some shortcomings. First, as a retrospective study, missing data is inevitable; for example, ECG results and arterial blood gas analysis data were not available in some children. Second, the number of cases was relatively small. A larger sample, multicenter, prospective study is needed.

## Conclusions

Compared with single *M. pneumoniae* infection, MPP coinfection with HAdV in children has a longer duration of fever, longer hospital stay, higher proportion of dyspnea, higher proportion of oxygen therapy and more severe disease status defined by the clinical score system.

## Data Availability

The datasets used and/or analysed during the current study are available from the corresponding author on reasonable request.

## Abbreviations

*M. pneumoniae*: *Mycoplasma pneumoniae*
CAP: Community-acquired pneumonia
ARDS: Acute respiratory distress syndrome
MPP: *Mycoplasma pneumoniae* pneumonia
HAdV: Human adenovirus
PCR: Real-time polymerase chain reaction
NCPAP: Noninvasive continuous positive airway pressure
PICU: Pediatric intensive care unit
CRP: C-reactive protein
PCT: Calcitonin
ALT: Glutamic-pyruvic transaminase
CK-MB: Creatine kinase isoenzyme
ECG: Electrocardiogram
IQR: Interquartile range
95% CI: 95% confidence interval
ECMO: Extracorporeal membrane oxygenation
RMPP: Refractory *Mycoplasma pneumoniae* pneumonia
